# Scratch and Wear Behaviour of Co-Cr-Mo Alloy in Ringer’s Lactate Solution

**DOI:** 10.3390/ma16072923

**Published:** 2023-04-06

**Authors:** Raimundo Silva, Marcos Dantas dos Santos, Rui Madureira, Rui Soares, Rui Neto, Ângela Aparecida Vieira, Polyana Alves Radi Gonçalves, Priscila Maria Sarmeiro M. Leite, Lúcia Vieira, Filomena Viana

**Affiliations:** 1 Department of Materials Engineering, University of the State of Amazonas (UEA), Darcy Vargas, Manaus 69050-020, Brazil; 2 Department of Metallurgical and Materials Engineering, Faculty of Engineering, University of Porto, Rua Dr. Roberto Frias, 4200-465 Porto, Portugal; fviana@fe.up.pt; 3 LAETA/INEGI—Institute of Science and Innovation in Mechanical and Industrial Engineering, Rua Dr. Roberto Frias, 4200-465 Porto, Portugal; 4 Department of Mechanical Engineering, Faculty of Engineering, University of Porto, Rua Dr. Roberto Frias, 4200-465 Porto, Portugal; 5 Department of Materials, Institute of Research and Development (IP&D), University of Paraíba Valley, São José dos Campos 12244-000, Brazil

**Keywords:** cobalt–chromium–molybdenum alloy, hip replacements, orthopaedics, scratch test, wear

## Abstract

Cobalt–chromium–molybdenum (Co-Cr-Mo) alloy is a material recommended for biomedical implants; however, to be suitable for this application, it should have good tribological properties, which are related to grain size. This paper investigates the tribological behaviour of a Co-Cr-Mo alloy produced using investment casting, together with electromagnetic stirring, to reduce its grain size. The samples were subjected to wear and scratch tests in simulated body fluid (Ringer’s lactate solution). Since a reduction in grain size can influence the behaviour of the material, in terms of resistance and tribological response, four samples with different grain sizes were produced for use in our investigation of the behaviour of the alloy, in which we considered the friction coefficient, wear, and scratch resistance. The experiments were performed using a tribometer, with mean values for the friction coefficient, normal load, and tangential force acquired and recorded by the software. Spheres of Ti-6Al-4V and 316L steel were used as counterface materials. In addition, to elucidate the influence of grain size on the mechanical properties of the alloy, observations were conducted via scanning electron microscopy (SEM) with electron backscatter diffraction (EBSD). The results showed changes in the structure, with a reduction in grain size from 5.51 to 0.79 mm. Using both spheres, the best results for the friction coefficient and wear volume corresponded to the sample with the smallest grain size of 0.79 mm. The friction coefficients obtained were 0.37 and 0.45, using the Ti-6Al-4V and 316L spheres, respectively. These results confirm that the best surface finish for Co-Cr-Mo alloy used as a biomedical implant is one with a smaller grain size, since this results in a lower friction coefficient and low wear.

## 1. Introduction

There is a great need for the development of new biocompatible materials for use in advanced surgical procedures, in order to meet the continually growing demand. The worldwide number of surgeries involving only the replacement of hard tissue is expected to exceed one million annually, demonstrating the importance of this issue and the scale of expenditure [1]. Several recent studies have investigated the use of new metal alloys based on steel [2], titanium [3], and cobalt [4] to replace bones in the human body.

Alloys based on cobalt and chromium have been employed in highly demanding medical applications [5], such as knee and hip implants, due to their excellent ability to maintain their mechanical properties and biocompatibility over long periods of use [6]. Hip joint replacement is one of the most successful orthopaedic surgeries [7]. These alloys have high creep resistance over extended periods, good stability at high temperatures, and excellent corrosion resistance in aggressive environments [8,9]. The importance of using cobalt–chromium–molybdenum alloys in the medical field is related to their physical characteristics, which are similar to those of the components they will replace (bone tissues and joints), as well as their ability to facilitate the regeneration, growth, and repair of bone tissues [8]. Furthermore, they present high resistance to corrosion in the physiological fluids of the body.

Therefore, cobalt–chromium–molybdenum alloys are among the most widely used materials for artificial joint implants, since they provide a good combination of mechanical properties and corrosion resistance [10,11,12]. It is necessary for these devices to possess the best possible mechanical properties and wear resistance in service [13], among other aspects, necessitating the search for new materials or the improvement of existing manufacturing processes. As pointed out by Gomes et al. [14], the microstructure of cobalt alloys allows for a higher concentration of carbon, with carbides dispersed inside the grains and around the margins, where their precipitation can provide the alloy with enhanced resistance and hardness. Typically, the microstructure of an as-cast cobalt-base alloy presents a dendritic α-FCC matrix that is metastable at ambient temperature, together with inter-dendritic precipitates composed mainly of M_23_C_6_ carbides [15]. Co-Cr-Mo alloys are manufactured via casting processes, sometimes being subjected to solution-annealing heat treatments and forging, and adopting different microstructures that impart hardness, strength, and wear resistance [10]. These microstructural changes affect the alloy’s mechanical and wear properties, while the microstructure and composition of the alloy affect its corrosion behaviour in body fluids, due to changes in surface chemistry [16,17,18,19]. In previous work [6], it was shown that modification of the investment casting process, using electromagnetic stirring (EMS) during solidification, resulted in a decrease in the size of the Co-Cr-Mo alloy grains, representing innovation in the manufacture of these alloys. However, it remains necessary to investigate whether there are associated improvements in the alloys’ mechanical properties (wear resistance and hardness). In addition, there are still no reports of the use of electromagnetic stirring for grain reduction in Co-Cr-Mo alloy used for implants.

A material used in load-bearing implants, such as hip and knee replacement prostheses, is simultaneously subjected to wear, due to the relative sliding movements of the surfaces of the joint components, and corrosion [20], due to exposure to aggressive body fluids [21]. Co-Cr-Mo alloys are considered corrosion-resistant, as a result of the oxide film formed on the metallic surface (1–4 nm thick), mainly containing Cr_2_O_3_, together with small contributions of Co and Mo oxides [22]. Furthermore, the abrasive wear behaviour is affected by the surface properties of the contacting materials [23]. Hence, further examination of the behaviour of the material under abrasion can contribute to the understanding of abrasive wear [24]. The Cr_2_O_3_ phase is a ceramic material that exhibits high corrosion and wear resistance, which are ideal qualities for orthopaedic replacement materials [25]. The detachment of wear particles is one of the main problems associated with total hip replacement (THR) implants [26]. The problems of friction and wear in the prostheses used to replace hip and knee joints mainly involve small-displacement wear (fretting wear) by metal-on-metal (MoM) contact, causing problems to the users of such prostheses [26,27,28,29]. The longevity of orthopaedic implants is affected by the formation of wear debris, which has been linked to problems such as tissue inflammation, bone loss, and implant loosening [12]. Wear reduces the useful life of prosthesis components, increasing replacement costs [18]. Wear mechanisms are typically classified as abrasive, adhesive, fatigue, fretting, erosive, or corrosive types [30,31]. The mechanisms of abrasion and adhesion are the most common in MoM contact, where abrasion occurs when a harder surface rubs against a softer surface, resulting in removal of the softer material. Adhesion wear occurs when two surfaces are pressed together under load, with the transfer of material from one surface to the other [32]. The metallic surface of the implant reacts with the air environment, forming a passive layer that acts as a barrier, separating the metal from the aggressive environment [33]. However, when in contact with the body fluids, this layer may be removed by corrosion or tribocorrosion, which can lead to the release of particles from the material [34,35].

Namus et al. [12], investigating the influence of crystal structure on the corrosion resistance of Co-based alloys, previously showed that a face-centred cubic phase exhibits better corrosion resistance than a hexagonal close-packed phase [12,36]. Although the resistance of Co-Cr-Mo alloys is affected by the carbon content, carbide distribution, and crystalline structure [37,38], grain size is also a feature that needs to be considered in efforts to improve the performance of these materials.

Therefore, the aim of the present research was to investigate the tribological behaviour of an as-cast Co-Cr-Mo alloy, where the grain size of the alloy was reduced via investment casting with electromagnetic stirring (EMS). The samples obtained were submitted to wear and scratch testing procedures, performed in a simulated biological fluid (Ringer’s lactate solution), using a tribometer system, to analyse wear and scratch.

## 2. Materials and Methods

### 2.1. Materials and Preparation

In this investigation, an ASTM F75 Co-Cr alloy (Zollern & Comandita, Maia, Portugal) was used. Table 1 presents its chemical composition, where the highest concentrations are chromium and molybdenum in a cobalt-base alloy. Samples of this alloy, which has a high carbon content, were produced via investment casting, with electromagnetic stirring during solidification, in order to reduce the grain size.

The alloy was melted in a vacuum furnace (INEGI, Porto, Portugal) equipped with high-frequency (50 kHz) induction heating in the melting chamber and electromagnetic stirring in the pouring chamber. The alloy load for casting and shell moulding was preheated for 4 h to 250 °C and 1000 °C, to avoid thermal shock when the alloy was filled into the mould.

The material was then rapidly transferred manually to the casting furnace. The ASTM F75 Co-Cr-Mo ingot (73 mm diameter and 160 mm height) was melted via induction heating, and the temperature was raised to 1500–1600 °C. The pouring of the Co-Cr-Mo alloy occurred at t = 373 s. In previous work, we simulated and produced the Co-Cr-Mo alloy. The time of pouring established in the simulation was 373 s, which was adopted for alloy production in the furnace [6].

After pouring, an electromagnetic field was applied to promote stirring of the melt, using electromagnetic field frequencies of 0, 15, 75, and 150 Hz. During the pouring and cooling steps, the alloy was kept under vacuum (0.1 mbar), reducing the possibility of a reaction between the alloy and the atmosphere (with oxide formation or hydrogen absorption). Twenty minutes after pouring, the casting and ceramic shell were removed from the vacuum furnace and cooled to room temperature. The ceramic shell was then cracked via a knockout process, and the cast sample was cleaned [6]. Increasing the EMS frequency resulted in a reduction in the grain size.

The average grain size was determined according to the ASTM E 112 standard [39]. This method consisted of drawing three lines on each sample and counting the number of grains intercepted by the test lines (N_c_). Then, the number of intercepts per unit length of each test line ($\bar{N}$_c_) and the mean linear intercept value for each test line ($\bar{l}$_i_) were calculated, as shown in Equations (1) and (2), where L is the test line length and M_f_ is the magnification. Finally, the average mean linear intercept value was calculated for each sample, in order to evaluate the grain size evolution.

(1) $$\overset{}{\underset{}{\bar{N_{c}}=}}\frac{N_{c}}{L/M_{f}}$$

(2) $$\overset{}{\underset{}{\bar{l_{i}}=}}\frac{1}{N_{c}}$$

After counting the grains, the number of grains for each sample was divided by the total area of the sample, as shown in Table 2.

**Figure 1:**
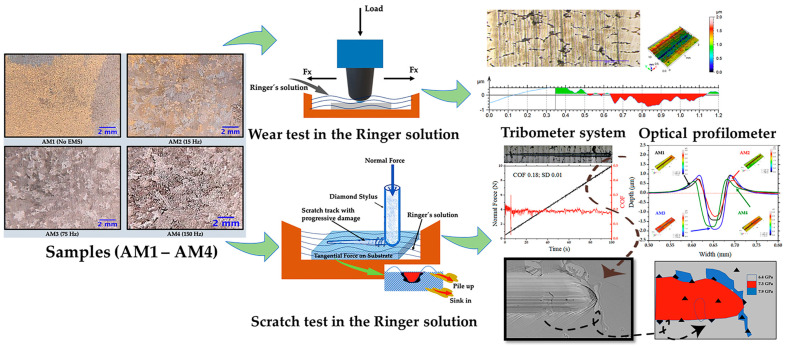
Scheme for tribological testing of the Co-Cr-Mo alloy samples.

Figure 1 shows the process for tribological characterization of the four Co-Cr-Mo alloy samples. The samples were produced using investment casting, together with electromagnetic stirring, to reduce their grain size. The samples were subjected to wear and scratch tests in simulated body fluid (Ringer’s lactate solution). Since a reduction in grain size can influence the behaviour of the material, in terms of resistance and tribological response behaviour the samples were compared using two spheres as counterbody, Ti6Al4V and 316L, in which was compared the friction coefficient, wear, and scratch resistance.

**Figure 2:**
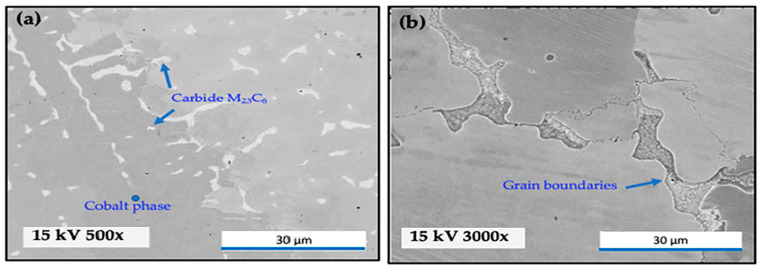
SEM images of polished surfaces of the Co-Cr-Mo alloy samples. (**a**) Dendritic structure, with an inter-dendritic carbide phase and (**b**) grain structure.

Figure 2 shows SEM images of the polished surfaces of the Co-Cr-Mo alloy samples. The images were used to evaluate the microstructure of the cast Co-Cr-Mo surface. Figure 2a shows a coarse dendritic structure characteristic shape, with an inter-dendritic carbide phase. The samples were rich in Cr and Mo, presenting a blocky morphology within the grains and at the grain boundaries. The grain structure was very coarse, with a large grain size, as shown also in Figure 2b. Similar structures were reported in other studies that used different production methodologies [10,17,40].

Co-Cr-Mo specimens with dimensions of 20.0 mm × 20.0 mm × 2 mm were wet-ground using 280–2500 grit SiC paper. Specimens were also polished using the polishing cloths during 10 min (until we obtained Ra = 0.03 μm). Four specimens (AM1, AM2, AM3, and AM4, produced using electromagnetic field frequencies of 0, 15, 75, and 150 Hz, respectively) were evaluated using wear and scratch tests. The mean values (friction coefficient, normal load, and tangential force) were calculated using the UMT micro-tribometer software, which recorded the normal and lateral forces (Fz and Fx, respectively) and the coefficient of friction (COF). The chemical and topographic characterisation of the wear tracks generated in the tribological tests employed SEM (20 keV accelerating voltage). The samples were cut and machined for wear and scratch testing. Sliding wear tests (using 316L steel and Ti-4Al6-V spheres as counterfaces) were performed with the samples immersed in Ringer’s solution. Scratch testing employed a diamond tip. SEM (Quanta 400 FEG ESEM, FEI, Frankfurt, Germany), optical microscopy (DM 4000 M, Leica Microsystems, Wetzlar, Germany), coherence correlation interferometry (CCI) using an optical profilometer (CCI-MP, Taylor Hobson, Leicester, UK), and indentation tests (Hysitron TI 950 Triboindenter, Bruker, Billerica, MA, USA) were used to characterise the wear track surfaces. Commercial Ringer’s lactate solution, purchased from Fresenius Kabi Brasil Ltd., was used to simulate normal physiological conditions or a similar-to-body environment. The Ringer’s lactate solution was composed of sodium chloride (0.6 g), potassium chloride (0.030 g), calcium chloride dihydrate (0.020 g), sodium lactate (0.310 g), and water to make up to 100 mL. Solar [41] reported that dilute NaCl-based solutions were satisfactory substitutes for studying the passive behaviour of metals. The use of Ringer’s solution is reported in the literature [35,42].

### 2.2. Wear Testing

**Figure 3:**
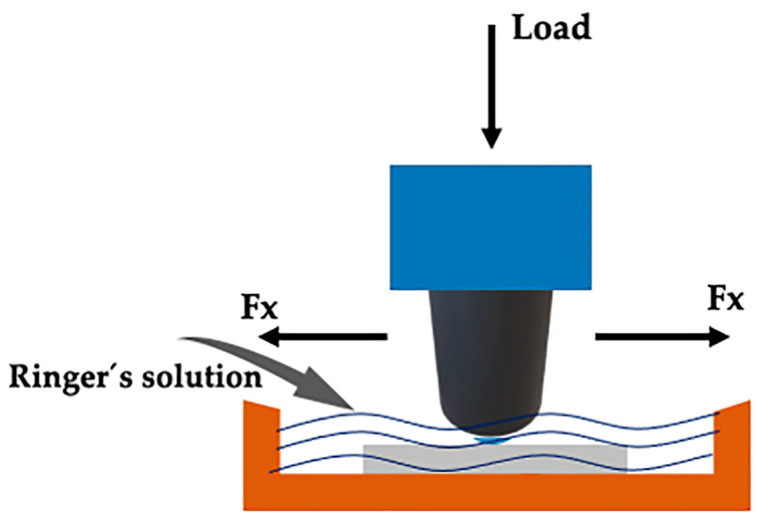
Schematic illustration of the UMT wear test device, with immersion of the sample in Ringer’s lactate solution.

Figure 3 Shows Schematic illustration of the UMT wear test device, with immersion of the sample in Ringer’s lactate solution. The sliding wear tests were performed using a UMT tribometer (CETR/Bruker, Coventry, UK), with reciprocal linear movement of the counterface (sphere) on the surfaces of the AM1, AM2, AM3, and AM4 samples, with a normal force (Fn) of 5 N (Hertzian initial contact pressure of 4.2 GPa), a duration of 1800s, and a speed of 20 mm s^−1^. The counterface materials were spheres with diameters of 4 mm, composed of Ti-6Al-4V or AISI 316L austenitic stainless steel, in order to vary the severity of the test. Three repetitions were performed with each sphere. As a substitute for bodily fluid, all the tests used Ringer’s lactate solution at body temperature (36 ± 2 °C). The sliding frequency was adjusted to 1 Hz and the stroke length was 10 mm. A container was designed to contain the sample and the Ringer’s lactate solution.

The specific wear coefficients (k) of the samples were calculated using the Archard equation:

(3) $$\overset{}{\underset{}{k=}}\frac{V}{W\times S}$$

where V is the worn volume, W is the load, and S is the sliding distance.

According to Bayer [43], there are normally three ways in which wear may be classified. One way is in terms of the appearance of the wear scar (pitted, spalled, scratched, polished, crazed, fretted, gouged, or scuffed). The second way considers the physical mechanism that removes the material or causes the damage (adhesion, abrasion, delamination, or oxidative damage). The third way considers the conditions of the wear situation (lubricated wear, unlubricated wear, metal-to-metal sliding wear, rolling wear, high stress sliding wear, or high-temperature metallic wear). In this work, lubricated wear was used for metal-on-metal and rolling wear.

### 2.3. Scratch Testing

The tribological behaviours of the Co-Cr-Mo alloy samples were investigated in scratch tests performed using a UMT tribometer, under wet conditions. The test parameters included a progressive increase from the preload of 0.5 N to 10 N in 1800 s, a scratch length of 10 mm, and a speed of 0.1 mm s^−1^, in accordance with the ASTM C1624-05 standard [44]. As stipulated in this standard, the scratch tests employed a diamond stylus (Rockwell C 120°) with a 200 μm tip radius, which was moved across the sample surface under a linearly increasing load. Both normal and tangential forces were applied, with the depth of the resulting scratch being continuously measured during the test. The surface topography and the scratch tracks were analysed using SEM. Coherence correlation interferometry (CCI) was used to assess the scratch track depth, wear volume, and pile-up.

### 2.4. Mechanical Characterisation

The Vickers microhardness of the alloy was evaluated according to the ASTM E-384-11 standard [45], using a Struers Duramin tester, with a load of 490.3 mN and a dwell time of 15 s.

The mechanical properties of the surface, including the hardness (H) and reduced elastic modulus (E_r_), were determined using the instrumented indentation technique, for each sample (AM1, AM2, AM3, and AM4). Firstly, the indentations were carried out using a Berkovich tip in a low load (maximum 10 mN) configuration. Calibration curves were performed before testing, according to the specifications of the triboindenter manufacturer. Machado et al. [46] reported that this procedure allows for calibration as a function of penetration depth in the range of 30 to 180 nm. The load–displacement curves obtained were evaluated using the Oliver and Pharr method to determine the indentation hardness (H_i_) [47,48]. The indentation analysis was performed using a Hysitron TI 950 Triboindenter instrument. In the indentation process, h_max_ is the maximum depth of penetration, h_f_ is the depth after loading, S = dL/dh allows for calculation of the stiffness of the material, and P_max_ is the maximum load applied. Hardness was calculated using Equation (4), where A(hc) is the contact area of the indenter with the sample during maximum loading.

(4) $$\overset{}{\underset{}{H=}}\frac{P_{\max}}{A(\mathrm{hc})}$$

In the nanoindentation test, the indenter was pushed into the surface of the sample, producing both elastic and plastic deformation of the material. Compared to macro- or micro-indentation tests, the difference when using nanoindentation machines is that the displacement (h) and the load (L) are continuously monitored with high precision [49]. The indentation procedure considered time intervals of 5 s, 5 s, and 2 s for loading, maintaining the load, and unloading the indentation tip, respectively. The experimental setup was used for evaluation of the properties of at least 21 indentations performed in different regions of each sample, and at least 48 indentations in different regions of the wear tracks.

As described by Machado et al. [46], the determination of H and E_r_ followed the Oliver and Pharr method [48]. The reduced elastic modulus (E_r_) of the Co-Cr-Mo alloy can be defined by Equation (5).

(5) $$\overset{}{\underset{}{\frac{1}{E_{r}}=}}\frac{\left(1-{v_{i}}^{2}\right)}{E_{i}}+\frac{\left(1-{v_{s}}^{2}\right)}{E_{s}}$$

where E_i_ (1140 GPa) and ν_i_ (0.07) are the elastic modulus and Poisson ratio of the diamond indenter, and E_s_ and ν_s_ are the elastic modulus and Poisson ratio of the Co-Cr-Mo alloy sample. The value of E_r_ was obtained from the nanoindentation tests, so E_s_ was calculated using Equation (5); It is also referred to as E, the elastic modulus of the Co-Cr-Mo alloy samples in this investigation. Walczack et al. [50] found that the Poisson ratio (ν_s_) is equal to 0.25 for casting.

### 2.5. Scanning Electron Microscopy and Profilometry

SEM and CCI were used to investigate the morphology of the Co-Cr-Mo alloy samples after the wear and scratch tests. The data evaluation and 3D visualisation allowed for determination of the shape of the craters produced from scratches and wear. As reported by Gahr [23], the relative fractions of the abrasion mechanisms of microcutting and microploughing can be described using the f_ab_ parameter, which is the ratio of the volume of material removed as wear debris to the volume of the wear groove [51]. In other words, it relates the area of the groove to the area of the ploughed material beside the groove, for a given scratch cross-section [24].

**Figure 4:**
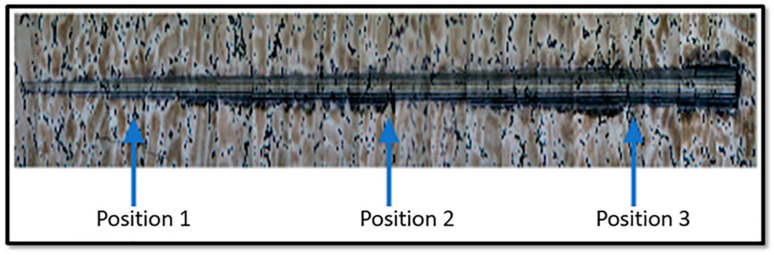
Determination of the scratch groove material removal factor (f_ab_) by evaluating three positions along the length of the scratch.

For the scratch and sliding tests, the f_ab_ material removal factor, given by Equation (6), was determined as shown in Figure 4, by evaluating 3 positions along the length of the scratch: the start (position 1), middle (position 2), and end of the track (position 3).

(6) $$f_{\mathrm{ab}}=\frac{A_{v}-(A_{1}+A_{2})}{A_{v\prime }}$$

**Figure 5:**
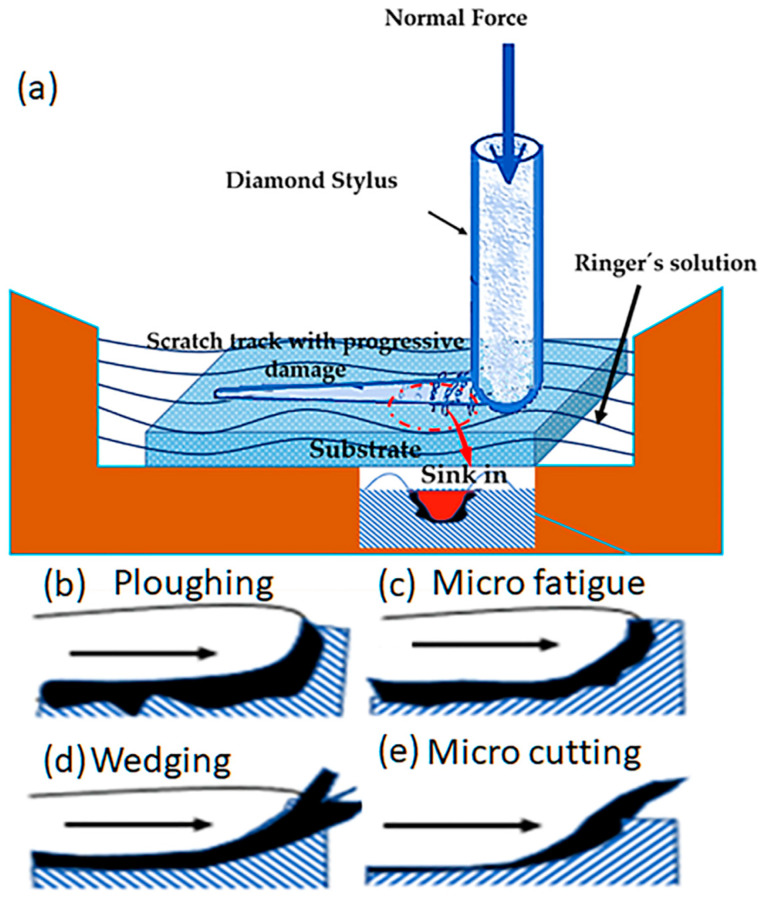
Schematic illustration of the theoretical model for calculating the ratio, using the f_ab_ material removal factor and the abrasive mechanisms: (**a**) ploughing, (**b**) microfatigue, (**c**) wedging formation, (**d**) microcutting, and (**e**) pile-up/sink-in. Crystallographic identification was performed using electron backscatter diffraction (EBSD) (FEI Quanta 400 FEG ESEM), in different regions of the samples, prior to the wear tests. The main objective was to identify the phases, carbide, and the alloy matrix.

Figure 5 shows a schematic illustration of the abrasive mechanism using a diamond stylus with the application of normal force. The scratch was produced by applying a progressive normal load from 0 to 5 N for all the samples. The images of the track were evaluated to identify mechanisms such as microploughing (Figure 5a), microfatigue (Figure 5b), wedging formation (Figure 5c), and microcutting (Figure 5d). All the mechanisms could produce pile-up, at the side of the track, or sink-in of material in the track, as shown in Figure 5e. The theoretical model was used to calculate the f_ab_ material removal factor, as described previously [52,53]. In Equation (6), A_v_ is the area of a wear groove, measured using a cross-section through the groove, and (A_1_ + A_2_) is the area of the material pushed to the groove edges via plastic deformation. It follows from this definition that ideal microploughing results in f_ab_ = 0 (with all the groove material being pushed to the sides) and ideal microcutting in f_ab_ = 1 (with the material being chipped out). Microcracking only occurs on brittle materials and leads to f_ab_ > 1 [23].

## 3. Results and Discussion

### 3.1. Surface Characterisation

There is a close relationship between grain size and wear resistance, as discussed by Chiba et al. [54]. For this reason, alteration treatments (such as recrystallisation) of the Co-Cr-Mo alloy structure are necessary for reduction of the grain size.

**Figure 6:**
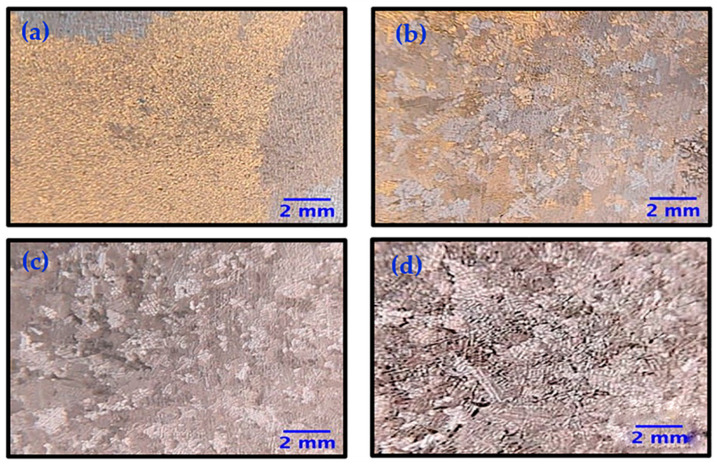
Optical microscopy images of the Co-Cr-Mo alloy samples, showing the grain size: (**a**) AM1 (coarse grains, no electromagnetic field application), (**b**) AM2 (fine grains), (**c**) AM3 (fine grains), and (**d**) AM4 (fine grains).

During the investment casting process, the samples were exposed to the action of a magnetic field (electromagnetic stirring, EMS) at the time of solidification of the alloy, with the aim of reducing the size of the grains and causing a morphological change in the casting dendrites. The samples were exposed to different magnetic fields (except sample AM1, for which no magnetic field was applied). Table 2 shows the applied frequencies and the grain sizes for each sample. Figure 6a shows an optical microscopy image for sample AM1, with no magnetic field applied, the measured grain size was 5.51 ± 1.91 mm. For samples AM2 Figure 6b, AM3 Figure 6c, and AM4 Figure 6d, which received applied magnetic fields of 15, 75, and 150 Hz, respectively, the grain sizes were 0.93 ± 0.67, 0.79 ± 0.54, and 0.84 ± 0.57 mm, respectively. Samples AM3 and AM4 presented the most significant reductions in average grain size, compared to sample AM1 (Table 2).

Table 2 shows the samples, EMS frequency, and average grain size. It can be seen that there was a correlation between the increase in EMS frequency and the decrease in grain size.

**Figure 7:**
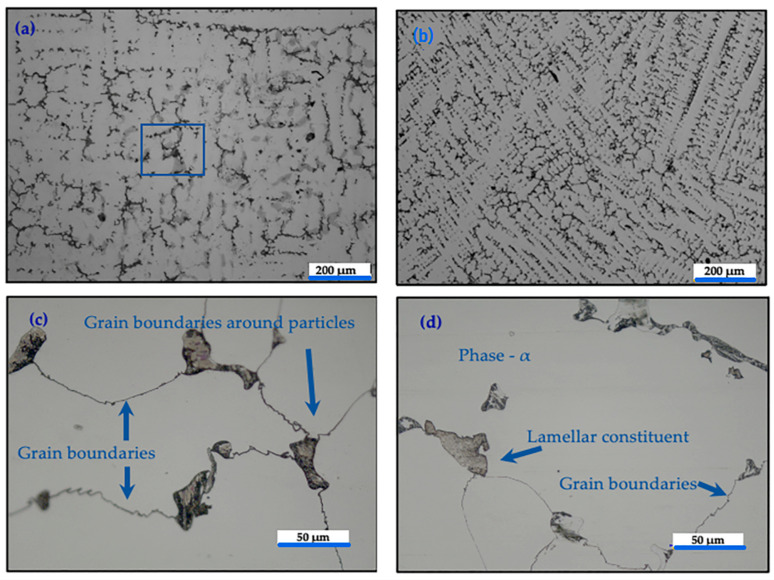
Optical microscopy images with two distinct scale bar 200µm and 50µm of the microstructure of the Co-Cr-Mo alloy: (**a**) cobalt-rich matrix phase, in blue square emphasis (AM1, no frequency applied); (**b**) abundance of M_23_C_6_ carbides (AM2, 15 Hz); (**c**) carbides formed at grain boundaries and thin grain contour lines (AM3, 75 Hz); (**d**) lamellar carbides phases at grain boundaries (AM4, 150 Hz).

Figure 7 shows optical microscopy images of the microstructures of the different Co-Cr-Mo alloy samples. It can be seen that large carbide particles were present at the grain boundaries. The most frequent carbides were M_23_C_6_ (M = Co, Cr, or Mo), and the percentage of carbon in the alloy was approximately 0.2%. Chromium is a carbide former, so most of the carbides in this alloy are rich in chromium, since it is the alloying element present at the highest concentration (approximately 29.9%). For sample AM1, without exposure to electromagnetic agitation, there was a lamellar structure around the carbide and grain boundaries (Figure 7a). For sample AM2, submitted to 15 Hz, there was high incidence of the secondary phase and a dendritic structure that presented a different orientation to the grains Figure 7b. For sample AM3 (75 Hz), Figure 7c shows the presence of dendritic structures, where the dark points are carbide, and the clear part is the matrix. Figure 7d, for sample AM4 (150 Hz), shows the lamellar constituent.

During solidification, dendrites of the gamma phase grow from the liquid, while the main alloying elements (C, Mo, and Cr) segregate into the liquid phase. The carbides nucleate in these enriched regions, forming a continuous layer.

**Figure 8:**
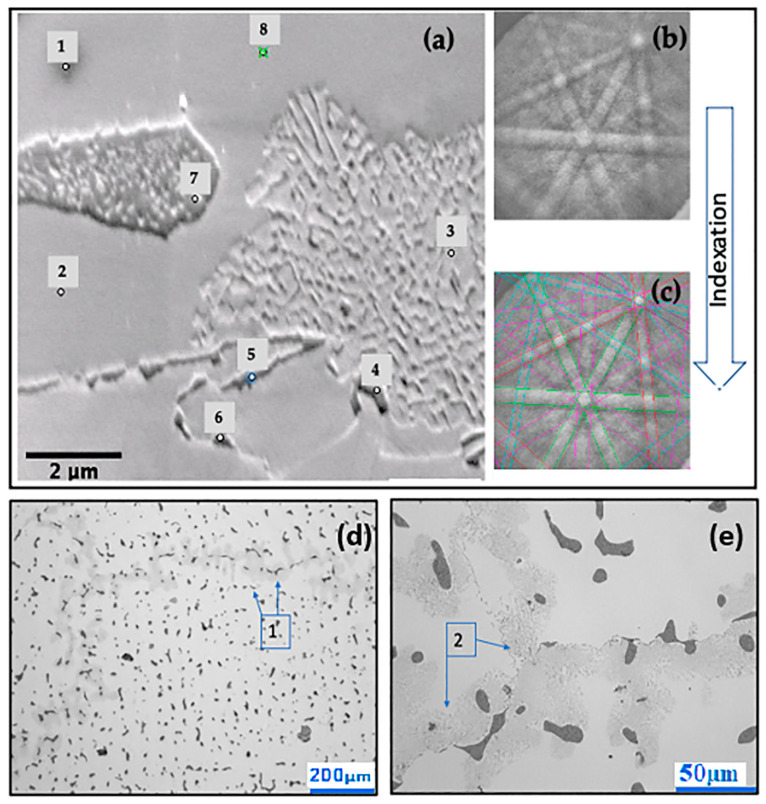
(**a**) SEM image showing the 8 points evaluated. (**b**) Kikuchi lines and the diffraction pattern. (**c**) Marking of higher lines. (**d**) Grain boundaries, identified as M_23_C_6_. (**e**) Lamellar constituent.

When a crystalline sample is bombarded by a beam of electrons inside a scanning electron microscope, the interaction between the electrons and the sample results in the emission of backscattered electrons, generating characteristic lines known as Kikuchi lines (or Kikuchi patterns) [55]. Each of these lines corresponds to the diffraction of electrons in certain crystallographic planes, as observed in Figure 8, described hereafter. 

Figure 8a shows a scanning electron microscopy image with eight points marked. Figure 8b shows the Kikuchi lines and the diffraction pattern for each point. Figure 8c shows the marking of higher-intensity lines and the indexation of the corresponding crystallographic planes at each point. Points 4, 5, 6, and 7 (thin dark plaques) were identified as M_23_C_6_ carbides. Points 1, 2, 3, and 8 were identified as a gamma Co phase with a face-centred cubic structure. The optical microscopy image presented in Figure 8d shows rounded particles (dark) in the inter-dendritic space along the grain boundaries, identified as M_23_C_6_ carbide and confirmed by the Kikuchi patterns (indicated by #1). Figure 8e shows the lamellar constituent within the boundaries of the grain (dark grey) (indicated by #2). 

Studies have found that the most prevalent carbides in cobalt matrix alloys are of the Cr_23_C_6_ type, and that this element, when in large quantities, actively participates in carbide formation [56,57,58]. The effect of carbides on wear is complex and depends on their nature, cohesion with the matrix, reactivity, and distribution [59]. The influence of the hard phases on sliding wear is determined by many metallurgical properties acting together, including hardness, ductility, and elasticity modulus, among others [23,60].

### 3.2. Wear Testing

**Figure 9:**
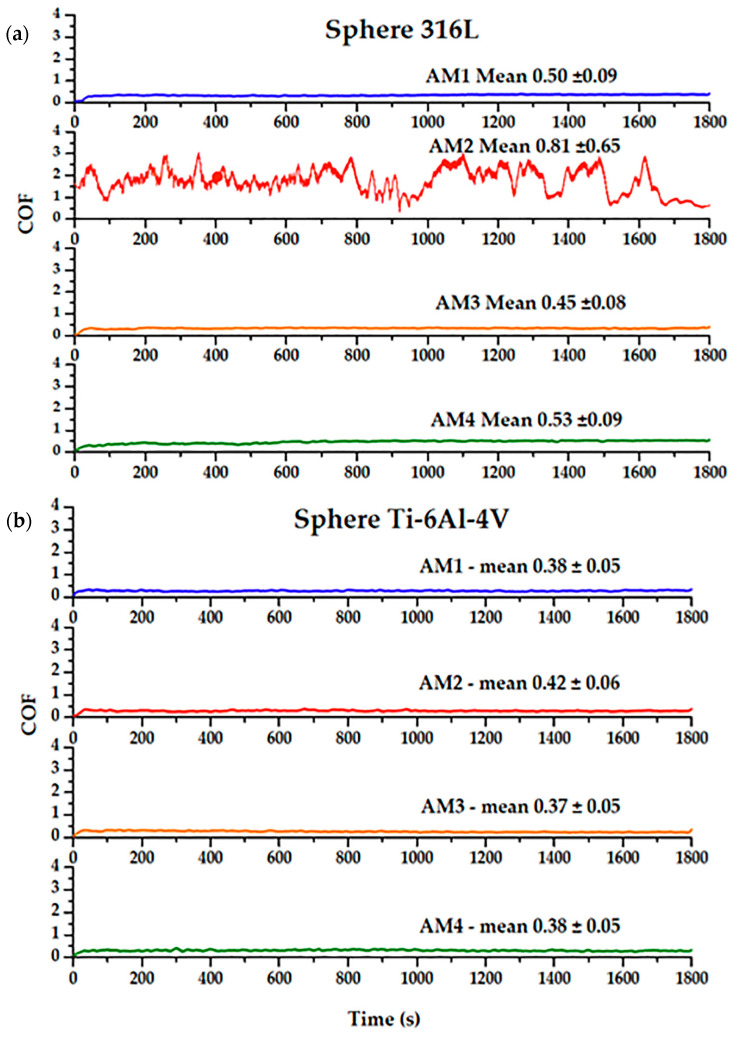
Friction coefficients variation from the Co-Cr-Mo alloy samples (AM1-AM4), and counterbody (**a**) a 316L steel sphere and (**b**) a Ti-6Al-4V sphere.

Figure 9 shows two plots comparing the friction coefficients obtained using the Co-Cr-Mo plate samples (AM1, AM2, AM3, and AM4) and the two types of counterface spheres 316L steel Figure 9a and Ti-6Al-4V Figure 9b. For each measurement was used a new sphere face.

The duration of the test was 1800 s in all cases. When the 316L sphere was used, the coefficients of friction were consistent and without significant fluctuations during the tests, with the exception of the AM2 sample, which presented a higher COF (0.81 ± 0.65), compared to the other samples. There was no obvious reason for this high value. The COF values obtained using the Ti-6Al-4V counterface showed no significant variation. For both spheres, the best COF corresponded to sample AM3 (μ = 0.37 and 0.45 for the Ti-6Al-4V and 316L spheres, respectively). The AM2 sample showed different behaviour, with the COF value increasing from 0.42 using the Ti6Al4V sphere to 0.81 using the 316L sphere.

Varano et al. [40] studied the influence of microstructure on cobalt alloy metal-on-metal wear in hip implants, under a boundary lubrication regime, where it was found that the wear was independent of the grain size. Chiba et al. [54] obtained higher COF values for cast and forged Co-Cr-Mo alloys (µ = 0.50–0.59 and 0.54–0.63, respectively), compared to the values obtained here for all the samples, except AM2. In the present work, better results were observed for the samples with smaller grains.

The worn surfaces of the cast Co-Cr-Mo samples showed differences in relief, as a result of the different phases present in the microstructure. The carbides at the grain boundaries were harder than at the interior of the grains, so they were less worn and appeared in relief at the wear track. This was probably related to the abrasive wear mechanism, using a sphere as a counterface. Abrasive grooves were also observed in these wear tracks.

**Figure 10:**
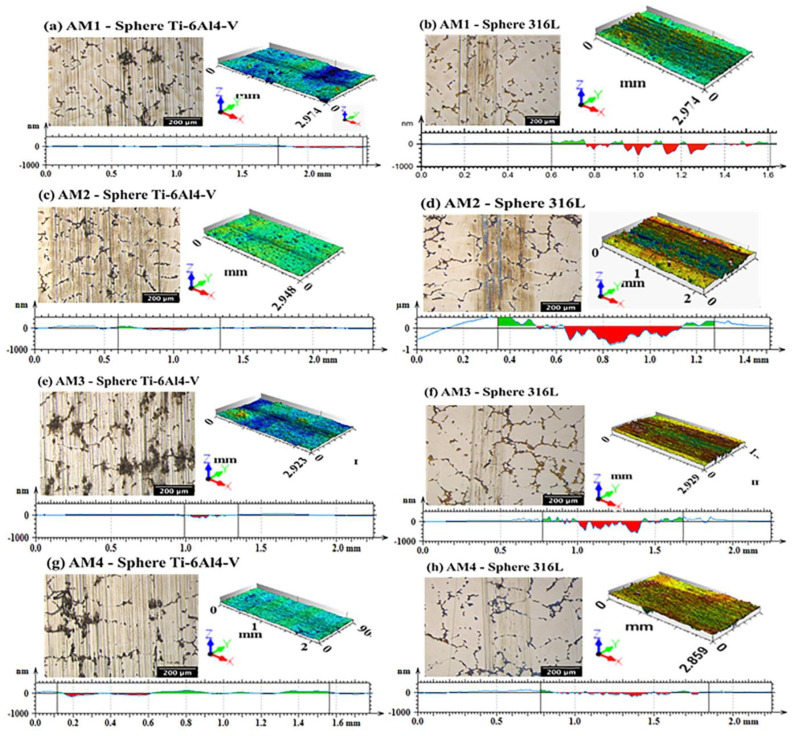
Images obtained from 3D profilometer analysis of eight cross-section wear tracks of Co-Cr-Mo samples AM1, AM2, AM3, and AM4, together with the corresponding depth profiles obtained using the interferometry technique (CCI).

As shown in Figure 10, the wear tracks on samples AM1, AM2, AM3, and AM4, using the Ti-6Al-4V sphere, showed an increase in their deformation lines, which is indicative of abrasive wear. When the 316L sphere was used, the deformation lines obtained for the same samples presented greater depth. The shallowest wear tracks were for sample AM4, which is indicative of greater wear resistance. For these tests, the samples were supported on a special tribological test stand. The results of all the friction tests for the Co-Cr-Mo alloy/316L and Co-Cr-Mo alloy/Ti-6Al-4V pairs are shown in Table 3.

The COF values for samples AM1, AM3, and AM4 were very similar, while higher values were obtained for sample AM2, most notably using the 316L sphere, indicating lower resistance to wear. As reported by Bayer [43] and Nass et al. [61], and as described in the ASTM procedure [62], geometric modification of the sample is caused by wear and the normal load, specifically in the contact region, due to three possible wear conditions: only sphere wear; only plate wear; and wear of both the sphere and the plate surfaces.

Figure 10 shows eight cross-section images of the wear tracks produced by the 316L steel and Ti-6Al-4V spheres on the different samples, obtained using a 3D profilometer, together with the corresponding depth profiles obtained using the interferometry technique. A comparison of the tracks produced by the Ti-6Al-4V sphere (Figure 10a,c,e,g) and the 316L steel sphere (Figure 10b,d,f,h) showed that the Ti-6Al-4V sphere caused less wear. The 316L steel sphere produced deep tracks on all the Co-Cr-Mo alloy samples.

As shown in Table 4, sample AM4 showed the lowest values for the wear rate and the worn volume (3.31 ± 0.9) × 10^−9^ mm^3^/Nm and (5.95 ± 0.85) × 10^−7^ mm^3^, respectively), as well as a low COF of 0.38. Balagna et al. [32] reported a friction coefficient of 0.35 ± 0.02 in wear experiments using a Co-Cr-Mo alloy pin and disc, which was close to the value found here using the Ti-6Al-4V sphere. In the same investigation [32], the authors found a wear rate of (2 ± 1) × 10^−7^ mm^3^/Nm for the same Co-Cr-Mo alloy pair. In this work, similar values were found for sample AM3, where the wear rate was (1.17 ± 1) × 10^−7^ mm^3^/Nm, using the Ti-6Al-4V sphere counterface, while the friction coefficient was 0.37 ± 0.05.

Table 4 shows a comparison of the grain sizes, mean COF, specific wear rate (mm^3^/Nm), and flat wear volume (mm^3^), measured during wear tests using the 316L and Ti-6Al-4V spheres against the Co-Cr-Mo alloy samples. The lowest flat wear volume was obtained for sample AM3, which had the smallest grain size. This confirmed the hypothesis that a smaller grain size led to a lower wear volume. Under slightly different conditions, Jiang and Stack [63] studied the sliding wear of the Stellite 12 (Co-Cr) alloy against 316 stainless steel, in an aqueous environment. It was found that the amount of wear on the steel samples increased dramatically under wet conditions, due to removal of the particles that formed a protective layer. The procedure they employed was very similar to that used here (a reciprocating system with total stroke length of 10 mm, a normal load of 5 N, and a body fluid solution). Duran et al. [64] studied the tribological properties of cast and selective laser melting (SLM)-processed Co-Cr-Mo alloys, under wet conditions (artificial saliva solution), using an alumina ball as the counterface. A wear rate of 2.27 × 10^−6^ mm^3^/Nm was reported for the cast alloy, which was higher than the values obtained in this work.

### 3.3. Scratch Testing

**Figure 11:**
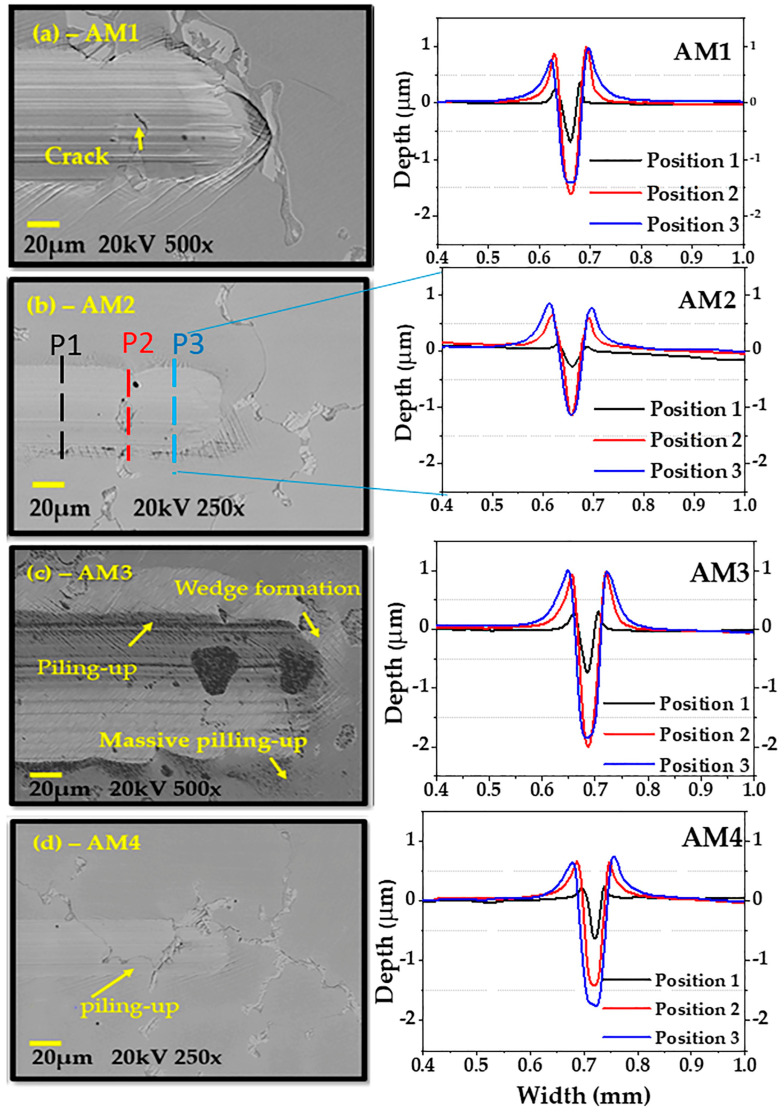
SEM images of the scratch grooves and profiles at different positions of the scratch tracks for samples (**a**) AM1, (**b**) AM2, (**c**) AM3, and (**d**) AM4.

SEM images and the wear profiles of the four Co-Cr-Mo alloy samples (AM1, AM2, AM3, and AM4) are shown in Figure 11. The samples were scratched with a diamond stylus, with a progressive load applied. The samples showed different deformation behaviours, including micro-abrasion. Cross-sectional views can be used to determine how the wear or depth of scratching occurred. The topographical image of the scratched strip shows only the upper surfaces with the grooves, cracking, and other defects. However, the wear track profile can indicate the amount of material lost, considering the subsurface area and the wear profile. The wear profiles for AM1, AM2, AM3, and AM4 were obtained at three positions (1 black line, 2 red line, and 3 blue line) along the length of the scratch test. Figure 11a shows that scratching of the AM1 sample led to massive pile-up (shoulders) next to the edges of the scratch. A volume of scratched material was pushed in front of the indenter, and was then ploughed to the sides or chipped out. Figure 11b shows scratches on the AM2 sample at low depth, together with displacement of the material due to the action of the diamond tip, at the end of the test. Figure 11c shows that scratching of the AM3 sample led to amounts of material near the edges of the scratch. A large volume of scratched material was pushed to the front of the indenter (wedge formation), with pile-up at the sides. Therefore, the abrasion mechanisms were microploughing and microcutting. Figure 11d shows scratching of the AM4 sample, with significant depth but no pile-up at the sides, while a small quantity of material was pushed to the front of the indenter. The scratch depth and alloy deformation behaviour were indicative of micro-abrasion mechanisms. It can be seen that a considerable volume of the scratched material that was pushed in front of the indenter was then ploughed to the sides or chipped out. Hence, the active abrasion mechanisms were microploughing and microcutting. A shallower scratch depth suggested better resistance to abrasion, with reduced loss of material. The average f_ab_ values for the samples are shown in Table 5. Over a longer scratch distance, the deformed volume could be ploughed out locally and concentrated at the sides, consequently leading to considerable pile-up, as shown in the profiles.

The ASTM Co-Cr-Mo alloy used by Mazzonello et al. [4] was subjected to scratch testing and showed plastic deformation, with accumulated material on the sides of the scar and in front of the indentation, but no cracking was observed in the scar. In the work by Balagna et al. [65], low-carbon (LC) and high-carbon (HC) samples of Co-Cr-Mo alloy were submitted to scratch testing, where the first instance of cracking appeared in the HC sample at around 6 N, and delamination phenomena occurred at around 25 N. Similar findings were observed in the present work. Pöhl et al. [24] also used scratch testing to analyse the behaviour of soft metallic materials. The softer the material, the greater the degree of pile-up formation and, consequently, the lower the proportion of the groove volume removed [7], as observed here for samples AM1 and AM3, which exhibited microploughing and pushing of the deformed material to the sides, resulting in substantial pile-up.

**Figure 12:**
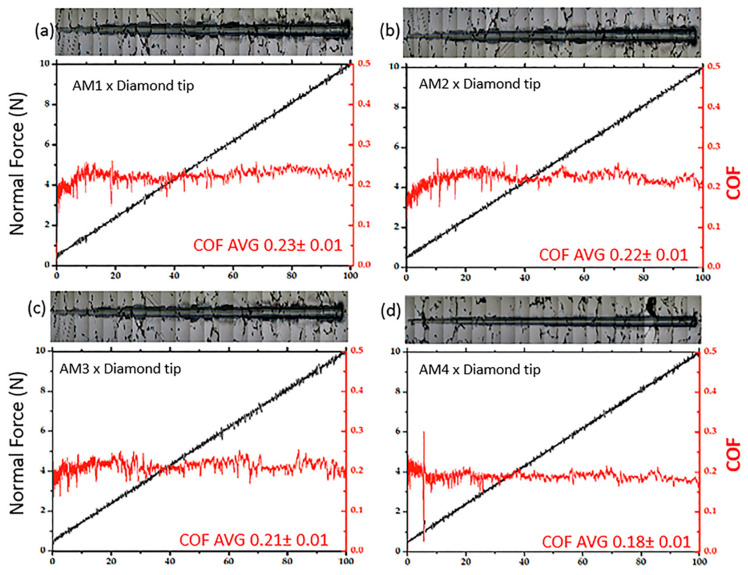
Experimental results for the ramp-load scratch testing of the Co-Cr-Mo alloy samples (**a**) AM1, (**b**) AM2, (**c**) AM3, and (**d**) AM4, using a Berkovich diamond indenter, showing the normal force (depth) and COF, together with images of the scratch tracks.

Figure 12 shows four plots for the ramp-load scratch testing using a Berkovich diamond tip and as scratched samples AM1-AM4. The normal force was ploted in black line and COF in red line, revealing considerable depth along the wear track diamond indenter, as showed in Figure 11. From Figure 12 is possible to observe the variation in the friction coefficient (COF) and the Fiction coefficient average that reflected the formation of material along the scratch. An optical microscope was used to record images of the scratch tracks, as shown above the graphs. 

The shape of the indenter influences the size and width of the pile-up, with more angular shapes producing narrow shoulders with greater peak heights, while more rounded shapes produce flatter shoulders [66]. In polycrystalline alloys, the grains have different crystallographic orientations relative to the surface plane, which significantly influences their behaviour during deformation. In other words, the deformation behaviour can be understood by the depth of the scratch obtained in the tests. Pile-up and chip formation can differ significantly from grain to grain, since the crystallographic orientation varies at the grain boundaries. The results for samples AM1–AM4 with different grain sizes (Table 2) showed that a larger grain size led to a greater tendency for pile-up. Although the Co-Cr-Mo alloy possesses some lamellar structures, it has a microstructure with a high quantity of carbides, causing greater surface hardness. However, the cobalt matrix tends to be softer and prone to substantial microploughing, with intense deformation. For AM1 (Figure 12a), the COF was 0.23 ± 0.01, resulting in low scratch resistance. For AM2 (Figure 12b), the COF was slightly lower (0.22 ± 0.02), and an increase in the removal of material was observed along the scratch, with pile-up and chip formation. The AM3 sample (Figure 12c), despite its COF of 0.21 ± 0.01, showed considerable removal of material, involving microcutting and microploughing from the start to the end of the scratch, and the presence of chipped-out material. Finally, the AM4 sample (Figure 12d) presented the lowest COF (0.18 ± 0.01) with low removal of material compared to the other samples, as can be seen by comparing the graphs and the scratch images (above the graph), with only two points where small amounts of material were removed by microploughing. For all the samples, the COF values showed quite low variation.

**Figure 13:**
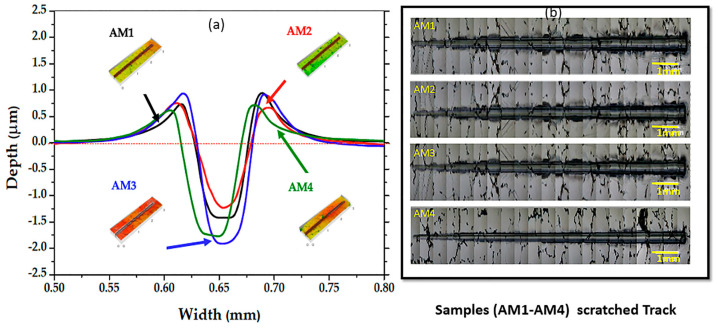
(**a**) Mean profile cross-section widths, as a function of depth, obtained via 3D optical profilometry. (**b**) Images showing the experimental results for the ramp-load scratch testing of Co-Cr-Mo samples AM1-AM4.

Figure 13a shows a plot of scratch profiles, while Figure 13b shows optical microscopy images of the total lengths of the scratch tracks obtained using the Berkovich diamond indenter. The track for sample AM3 shows greater depth, volume, and area, compared to the other samples. The tracks for samples AM1 and AM2 show high volumes and worn areas, while the track for sample AM4 shows a low volume and worn area (Table 5), reflecting the greater resistance of this sample during the scratch test.

Considering the pile-up formation, the shoulder area was significantly higher in position #3 (at a load of around 10 N). The pile-up area is not associated with mass removal, since it occurs at the edges of the ploughing area. The pile-up area should be discounted from the total area removed in order to calculate the scratch wear [67]. During the scratch, the samples generally maintained a COF above 0.18, although a lower value was obtained for sample AM4, as shown in Figure 12d. During scratching tests, the material is pushed in front of the conical stylus and is displaced to the edges of the grooves. With a greater load, the scratch depth increases and the volume of material that is horizontally displaced also increases, as described previously by Bellemare et al. [68].

Mischler and Muñoz [69] considered a value of E (elastic modulus) of 248 GPa for a Co-Cr-Mo alloy. More recently, O’Toole et al. [70] reported that a Young’s modulus of 210 GPa was far higher than that for a human leg or arm cortical bone, for which the Young’s modulus is less than 20,000 MPa [71]. Comparing the values found in this work with previous studies, the Young’s modulus of this alloy can be considered safe for implant applications. The results obtained for f_ab_, hardness, reduced elastic modulus, elastic modulus, plasticity index, track depth, track area, and worn track volume are displayed in Table 5 for the different Co-Cr-Mo alloy samples.

### 3.4. Mechanical Characterisation Results

The hardness-to-elastic modulus ratio (H/E) effectively indicates the ability of a surface to resist mechanical failure or damage during use [72,73]. However, while increased hardness of the surface implies an improvement in resistance to plastic deformation, fracture toughness may be reduced. The plasticity index (H/E) values for the samples are shown in Table 5. Qin et al. [74] reported a hardness (H) value of 7.9 GPa, an elastic modulus (E) of 178.5, and a plasticity index (H/E) of 0.044 for Co-Cr-Mo alloy, which are similar values to those obtained in the present investigation. Qin et al. [74] found strong evidence that the elastic modulus has an important influence on wear behaviour. Plastic deformation is reduced in materials with high hardness (H) and low elastic modulus (E_r_). Generally, low E_r_ is desirable, since it allows the applied load to be distributed over a larger area [75]. Leyland and Matthews [76] reported that the plasticity index is usually a good indicator of wear resistance. The present results (Table 5) showed higher H/E values for samples AM1, AM2, and AM4, indicating a higher capacity of the alloys to withstand external loads.In the work of Pöhl [24], it was reported that the stress in the test can lead to the occurrence of slip dislocation, depending on the orientation of the material structure. 

**Figure 14:**
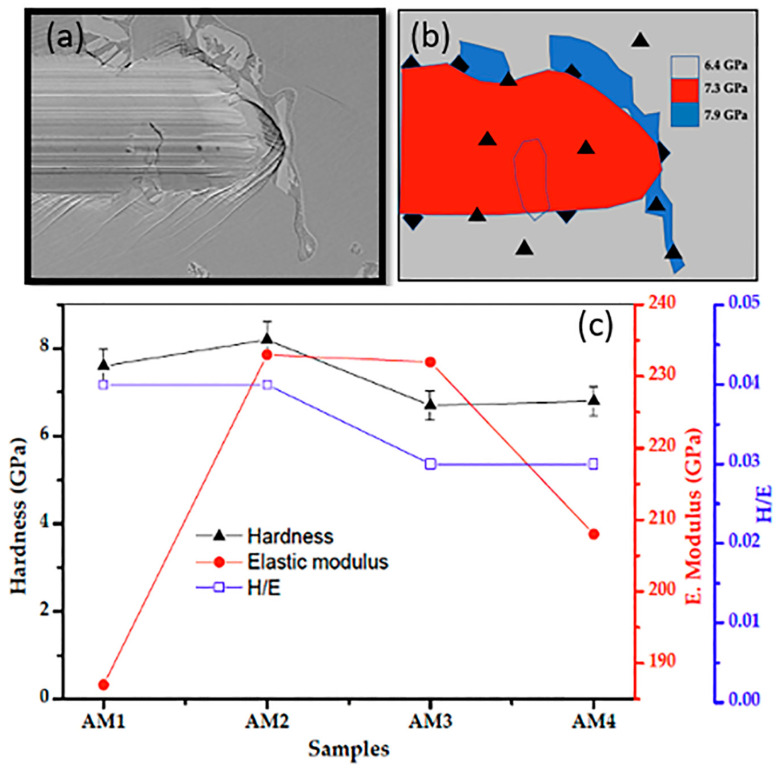
Results obtained using the Berkovich indentation technique. (**a**) Image of the end of the scratch performed on the surface of the sample. (**b**) Schematic interpretation of the indentation test in the sample (**c**) Hardness Hardness test results, Young’s modulus, and H/E ratio.

Figure 14a shows the an image from the end of the scratch performed on the sample surface using a Berkovich diamond tip. Additionally, the crystallographic orientation of the scratched Co-Cr-Mo alloy grains is shown, which influences the deformation behaviour of the material. There may be different deformation behaviours, such as pile-up and chip formation, related to the depth of the scratch at different grains along the track. 

Figure 14b provides a schematic interpretation of the image in Figure 14a, showing the indentation matrix the black triangles refer to the position were made the nanoindentation. From Figure 14b using the matrix is possible to observe that the nanoindentation was run out inside, in the boundary of the track, and outside of the track. 

The nanoindentation were runout usising an noindenter system with a (Rockwell C 120°) diamond tip radius of 200 μm. Figure 14c shows the hardness (H), reduced Young’s modulus (E_r_), and H/E_r_ ratio values obtained for the samples, which can be compared with data reported in the literature. The hardness (H) values obtained for samples AM1, AM2, AM3, and AM4 were 7.2 ± 1.31, 8.2 ± 1.04, 6.6 ± 0.42, and 6.8 ± 0.55 GPa, respectively (Table 5). These were very similar to the value of 9.4 ± 2.0 GPa reported for Co-Cr-Mo alloy by Moharrami et al. [77]. However, Martinez-Nogues et al. [78] found that the highest value was 5.87 ± 0.5 GPa for a forged sample, followed by values of 4.80 ± 0.5 GPa for an as-cast sample, 4.41 ± 0.3 GPa for an as-cast single thermal treatment sample, and 4.31 ± 0.2 GPa for an as-cast double thermal treatment sample. In the work by Balagna et al. [65], the values reported for Co-Cr-Mo alloys were 9 ± 1 GPa (wrought low-carbon alloy), 9 ± 1 GPa (cast low-carbon alloy), and 12 ± 1 GPa (cast high-carbon alloy). Higher hardness values were obtained for the alloy produced using this new process (EMS), even without thermal treatments.

A limitation of this investigation was that the bench tests did not replicate the biomechanics of a MoM hip joint in the human body. However, the main purpose of the scratch and wear tests was to qualitatively demonstrate the potential of the Co-Cr-Mo alloy produced using a new foundry process.

## 4. Conclusions

This investigation focused on the tribological behaviour of a Co-Cr-Mo alloy, fused using electromagnetic stirring to modify its microstructure and reduce its grain size from 5.51 mm (AM1) to 0.93 mm (AM2), 0.79 mm (AM3), and 0.84 mm (AM4). The samples were submitted to scratch testing in simulated body fluid, using 316L steel and TI-6Al-4V spheres as counterfaces. The wear resistance was improved, probably influenced by the microstructure and the number of secondary phases (carbides). The AM3 sample, with the smallest grain size (0.79 mm), presented the lowest friction coefficient (0.37 ± 0.05), which was consistent with the lowest plasticity index obtained for this sample. The results suggest that the reduced elastic modulus and plasticity index influenced the low friction coefficient for sample AM3. A third body formed during the friction tests could have contributed to the reduction in the friction coefficient. In addition, the lowest wear rate (3.31 ± 0.9) × 10^−^^9^ mm^3^/Nm) was observed for the AM4 sample, which also presented the lowest worn volume ((5.95 ± 0.85) × 10^−^^7^ mm^3^) (Table 4). Tribological performance testing of the four alloys showed that the wear rate of the AM4 sample was more than 10 times lower compared to the other three alloy samples (AM1, AM2, and AM3). In the scratch tests, Co-Cr-Mo alloy samples AM1, AM2, and AM3 showed greater pile-up deformation on both sides of the track. The Co-Cr-Mo alloys with reduced grain size, especially sample AM4, showed significant resistance in the scratch tests. The results obtained in these tests indicate that the method in which EMS is employed can be considered a promising route for the production of cobalt-based orthopaedic alloys, which should be explored in future research.

## Figures and Tables

**Table 1:** Chemical composition (wt%) of the studied Co-Cr-Mo alloy.

Element	Content
Cr	27–30%
Mo	5–7%
Ni	<0.5%
Fe	<0.75%
C	<0.35%
Si	<1.0%
Mn	<1.0%
W	<0.2%
P	<0.02%
S	<0.01%
N	<0.25%
Al	<0.1%
Ti	<0.1%
B	<0.01%
Co	Bal.

**Table 2:** Influence of EMS frequency on grain size.

Samples	EMS Frequency (Hz)	Grain Size (mm)
(a) AM1	No frequency	5.51 ± 1.91
(b) AM2	15	0.93 ± 0.67
(c) AM3	75	0.79 ± 0.54
(d) AM4	150	0.84 ± 0.57

**Table 3:** Mean COF values obtained in the reciprocating sliding tests for the Co-Cr-Mo alloy samples. Additionally, the grain sizes of the samples are shown.

Friction Coefficient (COF)			
Sample Co-Cr-Mo Alloy	Reciprocating Mode Sphere (ϕ = 4.0 mm) 5 N Normal Load, 10 mm Wear Track, 1.0 Hz Frequency		Grain Size
Tribological Pairs	Sphere	Sphere	
	316L steel	Ti-6Al-4V	(mm)
AM1/316L and Ti-6Al-4V	0.50 ± 0.09	0.38 ± 0.05	5.51 ± 1.91
AM2/316L and Ti-6Al-4V	0.81 ± 0.65	0.42 ± 0.06	0.93 ± 0.67
AM3/316L and Ti-6Al-4V	0.45 ± 0.08	0.37 ± 0.05	0.79 ± 0.54
AM4/316L and Ti-6Al-4V	0.53 ± 0.09	0.38 ± 0.05	0.84 ± 0.57

**Table 4:** COF, specific wear rate, and worn volume measured during wear tests using 316L and Ti-6Al-4V spheres against the Co-Cr-Mo alloy samples.

Sample and Sphere	Mean Friction Coefficient (COF) ± SD	Wear Rate (k) (mm^3^/Nm)	Wear Volume (mm^3^)	Grain Size (mm)
AM1_316L	0.50 ± 0.09	(6.44 ± 1) × 10^−7^	(1.16 ± 1) × 10^−4^	5.51 ± 1.91
AM1_Ti-6Al-4V	0.38 ± 0.05	(3.90 ± 1) × 10^−7^	(7.02 ± 1) × 10^−5^	
AM2_316L	0.81 ± 0.65	(1.40 ± 0.91) × 10^−5^	(2.53 ± 0.7) × 10^−3^	0.93 ± 0.67
AM2_Ti-6Al-4V	0.42 ± 0.06	(4.41 ± 1) × 10^−7^	(7.95 ± 1) × 10^−5^	
AM3_316L	0.45 ± 0.08	(7.83 ± 0.9) × 10^−7^	(1.41 ± 0.5) × 10^−4^	0.79 ± 0.54
AM3_Ti-6Al-4V	0.37 ± 0.05	(1.17 ± 1) × 10^−7^	(2.10 ± 1) × 10^−5^	
AM4_316L	0.53 ± 0.09	(4.95 ± 1) × 10^−8^	(8.90 ± 1) × 10^−6^	0.84 ± 0.57
AM4_Ti-6Al-4V	0.38 ± 0.05	(3.31 ± 0.9) × 10^−9^	(5.95 ± 0.85) × 10^−7^	

**Table 5:** Results obtained for Co-Cr-Mo alloy samples AM1, AM2, AM3, and AM4 submitted to the scratch testing procedure.

Sample	Material Removal Factor (f_ab_)	Hardness (H, GPa)	Reduced Elastic Modulus. (E_r_, GPa)	Elastic Modulus (E, GPa)	Plasticity Index (H/E)	Mean Scratch Depth (μm)	Scratch Track Area (μm^2^)	Worn Track Volume (μm^3^)
AM1	0.26	7.2 ± 1.31	187	210	0.03	1.43	19.40	7.3 × 10^−2^
AM2	0.51	8.2 ± 1.04	213	245	0.03	1.23	13.75	1.2 × 10^−2^
AM3	0.21	6.6 ± 0.42	232	273	0.02	1.93	25.34	1.5 × 10^−1^
AM4	0.32	6.8 ± 0.55	208	238	0.03	1.75	6.20	2.1 × 10^−2^

## Data Availability

Not applicable.
